# Modified Method of Scalp Fixation Promotes Survival of Complete Scalp Avulsion Replantation

**DOI:** 10.1002/ccr3.9665

**Published:** 2024-12-12

**Authors:** Lei Xu, Guangliang Zhou, Wen Ju, Yujun Zhang, Qianheng Jin, Lei Li, Ruixing Hou, Jihui Ju

**Affiliations:** ^1^ Suzhou Ruihua Orthopedic Hospital Suzhou Jiangsu China

**Keywords:** case report, complete scalp avulsion, microsurgical replantation, scalp fixation

## Abstract

A complete scalp avulsion injury is rare but extremely challenging to treat. Because of the dense blood vessel bundles in the scalp, replantation failure may occur if there is a subcutaneous hematoma. The method of scalp fixation is crucial for replantation to be successful. We successfully treated a total scalp avulsion injury using a modified scalp fixation method, coupled with microsurgical anastomosis, to avoid subcutaneous hematoma effectively and achieved a satisfactory outcome with a follow‐up of 9 years postoperatively.

## 1 Introduction

Full‐scalp avulsion injuries are often caused by hair being caught in rapidly rotating machinery, frequently accompanied by extensive skin loss and even exposure of the cranial bones. The scalp comprises dense hair follicles, a thick dermis, and nerves and blood vessel bundles. Significant blood loss often occurs in cases of full‐scalp avulsion injury, which can be life‐threatening in severe cases. In recent years, advancements in microsurgery have provided new approaches for treating complete scalp avulsion injuries [1, 2]. In some cases, the success of microsurgical replantation can be negatively affected by subcutaneous hematoma or inadequate drainage.

Most of the literature on scalp replantation has focused on vascular management [3, 4], with few addressing the scalp's fixation in replantation. Replantation failure may occur if there is a subcutaneous hematoma or inadequate drainage because of the dense blood vessel bundles in the scalp. In addition to efficiently preventing scalp separation from subcutaneous bruising, suturing the scalp to the periosteum can stop bleeding by compression and increase the survival probability during scalp replantation. In this study, the authors present a technique utilizing a spiderweb‐like suturing method to secure the scalp, effectively preventing subcutaneous hematoma formation and promoting successful replantation of total scalp avulsion injuries and achieves satisfactory results with a follow‐up of 9 years postoperatively.

## 2 Case History

**FIGURE 1:**
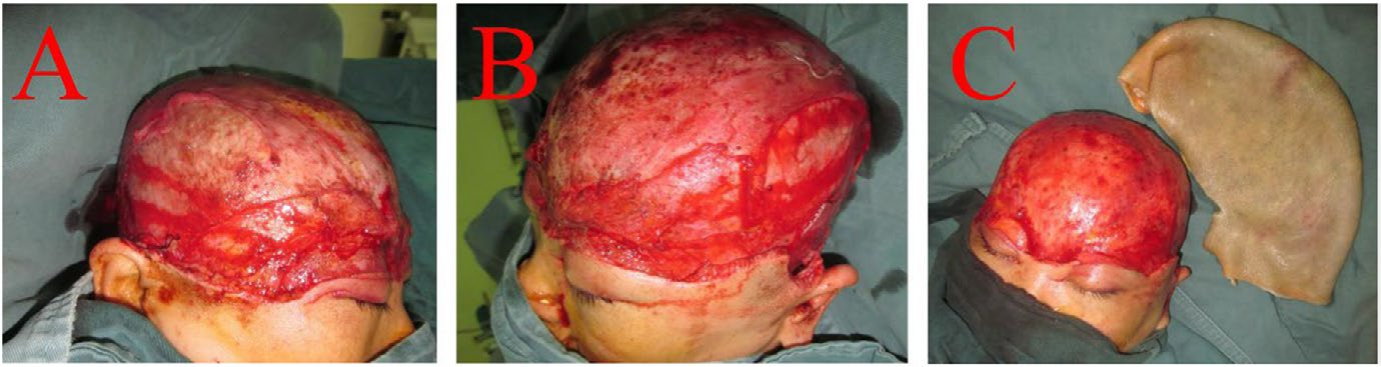
Complete scalp avulsion.

A 36‐year‐old female patient suffered a total scalp avulsion injury because of her hair getting caught in the rotating shaft of a machine. The scalp is avulsed from the galea aponeurotic, anteriorly extending from the nasion, the mid‐eyebrows, and lower eyelids bilaterally, posteriorly to the occipital scalp, laterally reaching the bilateral auricular roots and zygomatic arches, with an approximate area of 35.0 × 40.0 cm. The left auricle is torn and severely abraded, with exposed frontal, parietal, occipital, and temporal bones (Figure 1). The patient was in shock upon admission and was promptly treated with bandage compression for hemostasis and anti‐shock therapy. After blood pressure stabilized, surgery was performed under general anesthesia. The patient was admitted almost 3.5 h after the trauma.

## 3 Methods

The surgery was conducted in two groups: one group managed the scalp by cutting the hair, disinfecting the scalp with iodine, trimming the skin edges, identifying and marking blood vessels, whereas the other group managed the skull by disinfecting the skin and wound with iodine and after cleaning the wound, carefully identifying the blood vessels, and marking the superficial temporal arteries, veins, and occipital subcutaneous veins on both sides for later use.

**FIGURE 2:**
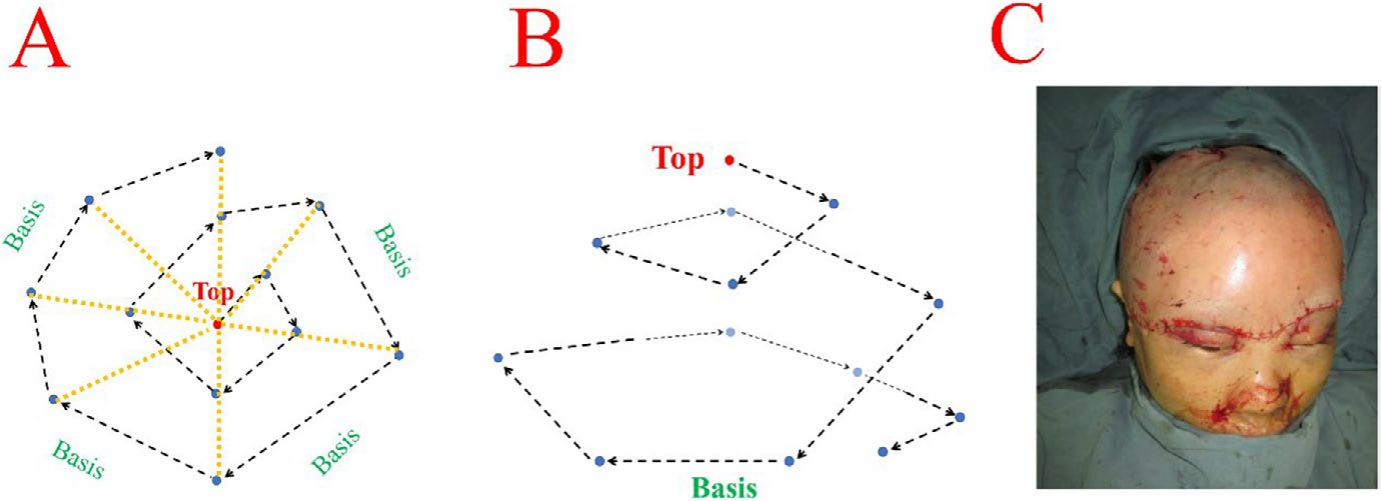
Scalp replantation. (A, B) Spiderweb suture technique diagram. (C) Postoperative appearance.

A spiderweb suture technique was used. Starting from the cranial vertex, the scalp was meticulously interrupted suture in place with absorbable sutures. The subcutaneous tissues on the left and right temporal sides were sutured and fixed to the periosteum (Figure 2A,B). Under the surgical microscope, the two arteries and four veins were delicately anastomosed using 10‐0 nontraumatic sutures, restoring blood circulation to the scalp. The subcutaneous tissues were sutured and fixed to the periosteum of the skull using 4‐0 absorbable sutures to ensure close approximation of the skin to the skull and prevent subcutaneous hematoma (Figure 2C). Drainage tubes were placed under the scalp and incision sites. Postoperatively, the drainage tubes were removed on the second postoperative day.

## 4 Conclusion and Results

**FIGURE 3:**
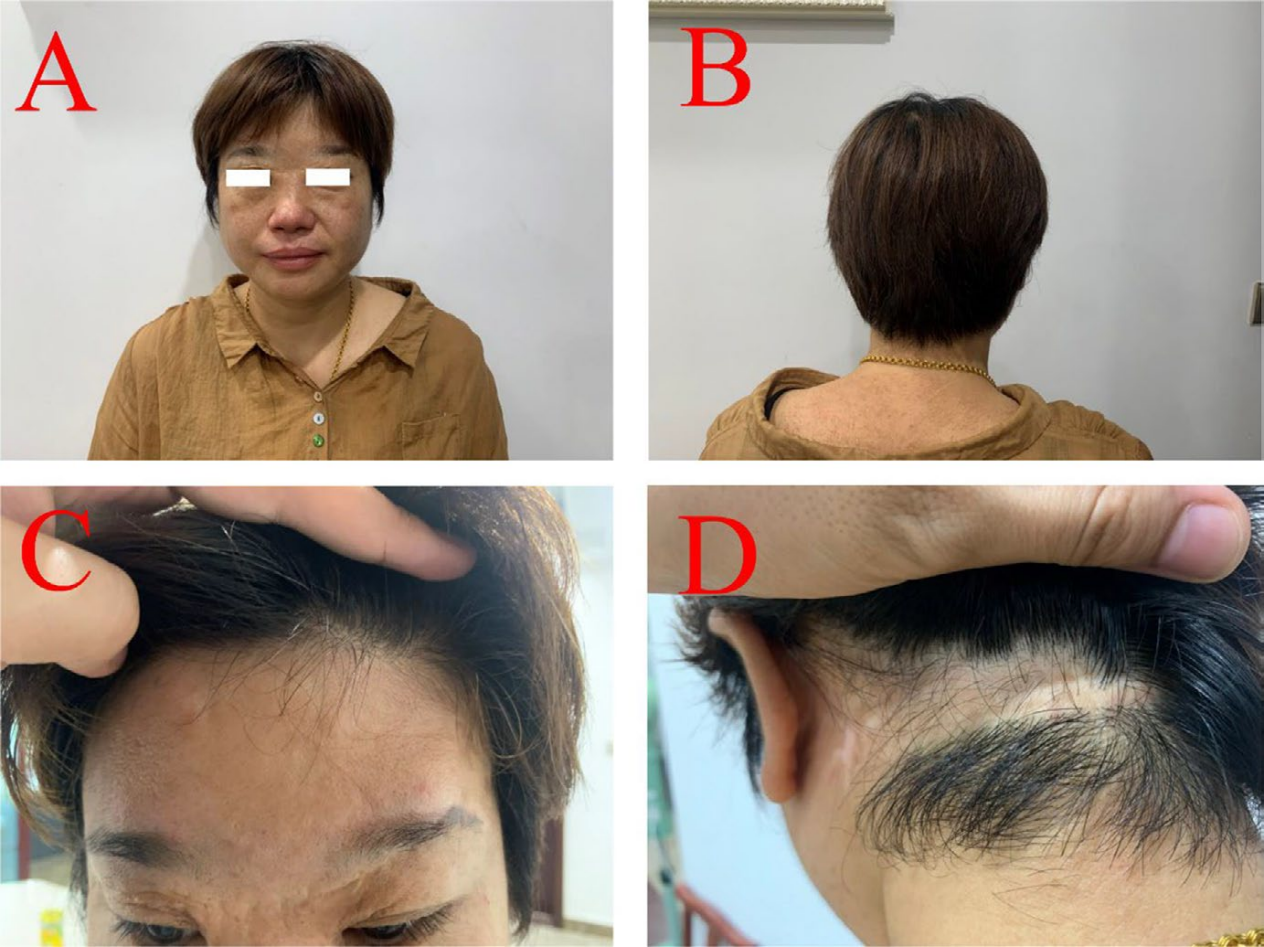
Nine years after transplantation. (A, B) Frontal and posterior aspects of the head. (C, D) The healing status of the frontal and posterior wounds.

Scalp replantation can achieve satisfactory results, and spiderweb fixation of the scalp can effectively prevent subcutaneous fluid accumulation, significantly improving the survival rate of scalp replantation. By the 10th day postoperatively, the scalp had fully healed. Viable scalp hair growth was observed and patients returned to normal activities 2 months postoperatively. After 7 months post‐surgery, the hair growth is dense, the eyelashes are growing well, and the protective sensation has returned. However, the scalp has occasional itching, and the right eye tends to tear up when looking at objects for a long time. Nine years after the surgery, there is satisfactory progress: the hair growth is lush and similar to the preoperative hair, the eyelashes are growing well, there is no local baldness, the scalp is soft, and the protective sensation is restored. Additionally, there is no obstacle to the opening and closing of the eyelids (Figure 3).

## 5 Discussion

Complete scalp avulsion is a rare and life‐threatening injury typically resulting from the entanglement of long hair in rotating machinery, such as agricultural or industrial equipment. Various treatment methods for scalp avulsion include thinning the scalp into medium‐thick skin flaps and replanting them in situ [5]. However, the success rate of this method is relatively low. For cases with exposed cranial bones, skull perforation can be performed, and skin grafting is done after granulation tissue formation. Another approach involves using the greater omentum for free transplantation to the head, with skin grafting on the greater omentum [6, 7]. However, these methods may result in alopecia deformities. With the advancement of microsurgical techniques, utilizing vascular anastomosis for full‐scalp replantation is considered the best method for treating scalp avulsion injuries. This approach ensures blood supply to the scalp and promotes hair regrowth, typically avoiding alopecia deformities.

The scalp receives a generous supply of blood from various arteries in the subcutaneous layer above the galea, including the superficial temporal, occipital, supraorbital, and supra‐auricular arteries. If postoperative drainage is inadequate, leading to the formation of a subgaleal hematoma, it can cause separation of the scalp and ultimately result in the failure of scalp replantation. Therefore, preventing scalp separation is key to ensuring scalp replantation's success. Currently, the literature merely touches on how to fix the scalp, with the main method being interrupted sutures, but the specifics of this fixation have not been reported in the literature. Lin et al. [8] reported the use of interrupted sutures for scalp fixation, but postoperatively, a balloonet is required to protect the replanted scalp and for conventional monitoring. Because of the large area of the scalp, there is a risk of scalp sliding, which may damage the anastomosed vessels and the formation of subcutaneous hematomas. In this work, we have refined the technique for fixing the scalp, presenting a novel method that secures the scalp sporadically using a spiderweb‐like design. First of all, spiderweb anchoring of the scalp efficiently prevents displacement of the scalp during or after surgery, which could lead to vascular rips, breaks, anastomotic distortion, and the production of large subcutaneous hematomas after surgery. It also makes intraoperative vascular anastomosis easier. Second, it makes it easier for the recipient site and the replanted scalp to come into close contact, which promotes the development of a shared blood supply. Finally, it lessens subcutaneous oozing, successfully stopping hematomas from forming under the scalp. As a result, the enhanced scalp fixation technique can significantly increase the success rate of replantation. The main drawback of this study is that it requires the full scalp to be in good condition, with minimal contusion of the avulsed skin, and the skin must be suitable for replantation.

## Author Contributions

**Lei Xu:** conceptualization, methodology, resources, writing – original draft. **Guangliang Zhou:** conceptualization, methodology, writing – original draft. **Wen Ju:** conceptualization, data curation, methodology. **Yujun Zhang:** conceptualization, methodology, resources. **Qianheng Jin:** investigation, methodology, resources, software. **Lei Li:** investigation, methodology, resources. **Ruixing Hou:** conceptualization, investigation, methodology, project administration, supervision, writing – review and editing. **Jihui Ju:** conceptualization, formal analysis, investigation, methodology, project administration, resources, supervision, writing – review and editing.

## Consent

Written patient consent has been signed and obtained from the patient.

## Conflicts of Interest

The authors declare no conflicts of interest.

## Data Availability

The data that support the findings of this study are available from the corresponding author upon reasonable request.
